# Impact of statin use on COVID-19 outcomes in hospitalized patients in Saudi Arabia: a retrospective cohort study

**DOI:** 10.25122/jml-2024-0371

**Published:** 2024-11

**Authors:** Omar Al-Nozha, Ahmed Abulkhair, Amal Hawsawi, Sawsan Sayed, Khlood Alrowathi, Nawaf Aldeeb, Hadel Alghabban, Ghaidaa Elmehallawy, Dalya Iskandarani, Mohammed Lhmdi, Inass Taha

**Affiliations:** 1 College of Medicine, Taibah University, Al-Madinah Al-Munawwarah, Saudi Arabia; 2 Ministry of Health, Al-Riyadh, Saudi Arabia; 3 King Salman Medical City, Ministry of Health, Al-Madinah Al-Munawwarah, Saudi Arabia; 4 Ministry of National Guard Health Affairs, Al-Madinah Al-Munawwarah, Saudi Arabia; 5 Bahcesehir University, Istanbul, Turkey; 6 Department of Critical Care, Saudi German Hospital, Al-Madinah Al-Munawwarah, Saudi Arabia

**Keywords:** COVID-19, statins, cardiovascular diseases, mortality, Saudi Arabia

## Abstract

There is an increasing requirement for new therapeutic approaches to address lung inflammation caused by COVID-19. Recent evidence suggests that statins may reduce mortality in patients with respiratory infections. This study aimed to investigate the impact of statin use on COVID-19 outcomes among hospitalized patients at Ohud Hospital and King Salman Medical City (KSMC) in Madinah, Saudi Arabia. A retrospective cohort study was conducted, including 547 patients with confirmed COVID-19 diagnoses admitted between March 2020 and December 2022. Patients were classified into statin and non-statin users based on statin administration during hospitalization. Logistic regression analyses—including univariate, multivariate, and predictive stepwise models—were employed to assess associations between statin use and clinical factors. Among the 547 patients, 200 (36.5%) were prescribed statins upon admission. Statin users were predominantly men and older. The presence of low-density lipoprotein (LDL) levels ≥ 100 mg/dL, cardiovascular disease (CVD), and advanced age were identified as strong predictors of statin use, with odds ratios (ORs) of 11.1, 3.8, and 3.1, respectively. Furthermore, the odds of receiving statins were significantly higher in male patients, individuals with hypertension, those with HbA1c levels ≥ 8%, and patients with positive cultures and sensitivity results. Statin use was associated with an 18%% reduction in the risk of mortality, with an adjusted OR of 0.80 (95% CI, 0.30–2.32), and a 7% reduction in the risk of hospital stay > 10 days, although these findings did not reach statistical significance. Among patients with COVID-19, LDL ≥ 100 mg/dl, CVD, and patients older than 60 years were identified as strong predictors for statin prescription.

## INTRODUCTION

The COVID-19 pandemic caused by the SARS-CoV-2 virus has resulted in over 108 million infections and more than 2.38 million deaths worldwide since early 2019 [1]. Despite advancements in vaccinations and management, COVID-19 continues to pose a significant threat to health. In addition to respiratory failure, patients with COVID-19 are at an increased risk of various complications such as thrombosis, cardiovascular issues, pulmonary embolism, deep vein thrombosis, and ischemic stroke [2]. The angiotensin-converting-enzyme 2 (ACE2) receptor plays a crucial role in allowing the virus to enter host cells. It is found in multiple organs, including the lungs, heart, blood vessels, brain, gastrointestinal tract, and kidneys. ACE2 is an important enzyme in the renin-angiotensin system, and its activity helps counteract the harmful effects of angiotensin II [3].

Researchers have been investigating cost-effective and practical treatments to enhance patient outcomes and decrease reliance on mechanical ventilation in hospital settings. One promising avenue is the use of statins, which are known to inhibit an enzyme called 3-hydroxy-3-methyl-glutaryl-CoA (HMG-CoA) reductase [4]. Statins have gained attention as potential adjunctive therapies against infectious diseases. In addition to antibacterial effects, particularly in preclinical tuberculosis models [4,5], statins have also demonstrated the ability to enhance the body's antiviral response in preclinical models, such as inhibiting human immunodeficiency virus (HIV) replication and reducing the viral load of cytomegalovirus and respiratory syncytial virus [6-9]. Moreover, statins improve the protection against influenza when used as a vaccine adjuvant. Previous studies have shown that statins can reduce the severity of influenza infections and decrease hospitalizations and deaths associated with viral pandemics [10-13]. These findings suggest that statins could be an adjunctive treatment for viral pandemics like COVID-19 [10,13]. All these effects are attributed to their impact on various molecular pathways in the body, including inhibition of mechanistic target of rapamycin complex 1(mTORC1) activation, activation of adenosine monophosphate-activated protein kinase (AMPK), increased nuclear translocation of transcription factor EB (TFEB), modulation of nuclear factor kappa-light-chain-enhancer of activated B cells (NF-κB) activity, and inhibition of protein prenylation [14-16].

Recent findings from a retrospective study conducted in an elderly care facility indicated that the administration of statins to residents diagnosed with COVID-19 was associated with milder symptoms and improved clinical outcomes [17]. Furthermore, several other studies have suggested a potential association between statin use and reduced mortality in COVID-19 cases [18-20]. Building on these findings, the current retrospective study aimed to investigate the impact of statin use on COVID-19 outcomes in hospitalized patients admitted to Ohud Hospital and King Salman Medical City in Madinah, Saudi Arabia.

## MATERIAL AND METHODS

### Study design and participants

This retrospective study included 547 confirmed cases of COVID-19 admitted to Ohud Hospital (a 250-bed referral quarantine hospital for COVID-19 cases) and King Salman Medical City (KSMC) in Madinah, Saudi Arabia between March 2020 and December 2022. Among these patients, 200 received in-hospital statin therapy (the statin group), while 347 did not receive statins during their hospitalization (the non-statin group). Patients were eligible for inclusion if they were 18 or older and had a confirmed COVID-19 diagnosis via polymerase chain reaction (PCR) testing. Only those admitted to the hospitals during the specified study period were included. Exclusion criteria included pregnant women, individuals less than 18 years old, and patients with contraindications to statin use.

### Data collection

Patient data were collected retrospectively from medical records, including demographic information (age, sex, and body mass index [BMI]), co-morbidities (hypertension [HTN], renal failure [RF], diabetes mellitus [DM], cardiovascular diseases [CVD], and lung disease), and laboratory parameters (D-dimer, hemoglobin [Hb], glycated hemoglobin [HbA1c], white blood cell count [WBCs], C-reactive protein [CRP], and low-density lipoprotein [LDL]). In addition, in-hospital outcomes such as intensive care unit (ICU) admission, mechanical ventilation use, hospital stay duration, and mortality rates were recorded.

### Statistical analysis

Statistical analyses were performed using SPSS 22.0 software (version 22.0 for Windows, SPSS Inc., Chicago, IL). Categorical variables were analyzed using the chi-square test, while continuous variables were analyzed using the student's *t*-test. A univariate logistic regression analysis was conducted to assess the association between statin use and demographic, co-morbidity, and laboratory variables. Variables found significant in univariate analyses (*P* < 0.05) were included in a multivariate logistic regression model to identify independent predictors of statin use. Finally, a multivariate logistic regression model was used to evaluate the impact of statin use on in-hospital outcomes, adjusting for potential confounders such as demographic characteristics, co-morbidities, and laboratory findings. The results of regression analyses were presented as odds ratios (OR) with 95% confidence intervals.

## RESULTS

A total of 547 patients with COVID-19 were included in the study, of whom 200 (36.5%) received statins upon admission. Statin users were significantly more likely to be men (75.5%% vs. 24.5%, *P* < 0.001) and older (64.2 ± 13.4 vs. 53.5 ± 18.2 years, *P* < 0.001) compared to non-statin users. The use of statins was also higher among patients with obesity (BMI ≥ 30 kg/m^2^) compared to normal and overweight, although not significant (Table 1).

**Table 1:** Demographic and anthropometric data of study groups

Demographic data^*^	Statin group (*n* = 200) n (%)	Non-statin group (*n* = 347) *n* (%)	*P* value
**Age in years**, mean ± SD	64.2 ± 13.4	53.5 ± 18.2	<0.001^**^
**Age in years** ≥ 60 < 60	76 (38.0) 124 (62.0)	224 (64.6) 123 (35.4)	<0.001^**^
**Sex** Men Women	151 (75.5) 49 (24.5)	189 (54.4) 158 (45.6)	<0.001^**^
**Body mass index (BMI)** < 30 kg/m^2^ ≥ 30 kg/m^2^	50 (25.0) 150 (75.0)	105 (30.3) 242 (69.7)	0.18

*Data are presented as mean ± SD or *n* (%). **Significant

Table 2 highlights the OR associated with statin use and laboratory factors among patients with COVID-19. Statin use was significantly higher in patients with HbA1c ≥ 8% (OR = 2.20, *P* < 0.001), LDL > 100 mg/dL (OR = 8.40, *P* < 0.001), and positive blood cultures (OR = 2.40, *P* < 0.001). Although not statistically significant, elevated odds of statin use were observed in patients with anemia (Hb < 12 g/dL), leukocytosis (WBC > 12 x 10^3^), and positive D-dimer results.

**Table 2:** The association between statin use and laboratory factors among patients with COVID-19

Laboratory variables	Statin group *n* = 200 *n* (%)	Non-statin group *n* = 347 *n* (%)	OR (95% CI)	*P* value
**CRP > 5.0 mg/L**	148 (74.0)	263 (75.7)	0.91 (0.61–1.36)	0.64
**D-dimer positive**	67 (33.5)	91 (26.2)	1.40 (0.97–2.06)	0.07
**Hb <12 g/dl**	48 (24.0)	67 (19.3)	1.30 (0.87–2.01)	0.20
**HbA1c ≥ 8%**	78 (39.0)	79 (22.7)	2.15 (1.48–3.17)	<0.001^**^
**WBCs > 12.X10^3^**	107 (53.5)	160 (46.1)	1.30 (0.95–1.91)	0.10
**LDL > 100 mg/dl**	152 (76.0)	95 (27.4)	8.40 (5.62–12.5)	<0.001^**^
**Positive blood culture**	84 (42.0)	70 (20.2)	2.40 (1.61–3.62)	<0.001^**^

CRP, C-reactive protein; Hb, Hemoglobin; WBCs, White blood cell count **Significant

The association between statin use and co-morbidity-related factors is presented in Table 3. Patients with CVD, hypertension, and diabetes mellitus were significantly more likely to be prescribed statins, with ORs of 5.52, 2.50, and 3.75, respectively (*P* < 0.001). Although the odds of statin use were higher among patients with renal failure (OR = 1.36) and among patients with lung disease (OR = 1.48), the results were not significant.

**Table 3:** The association between statins use and co-morbidity-related factors

Co-morbidities	Statin group *n* = 200 *n* (%)	Non-statin group *n* = 347 *n* (%)	OR (95% CI)	*P* value
**Renal failure**	88 (44.0)	127 (36.6)	1.36 (1.01-1.94)	0.09
**CVD**	97 (48.5)	51 (14.7)	5.52 (3.67-8.29)	<0.001^**^
**Hypertension**	128 (64.0)	149 (42.9)	2.50 (1.74-3.60)	<0.001^**^
**Diabetes mellitus**	145 (72.5)	143 (41.2)	3.75 (2.57-5.49)	<0.001^**^
**Lung disease**	30 (15.0)	37 (10.7)	1.48 (0.88-2.48)	0.14

CVD, cardiovascular disease **Significant

The predictors of statin use were examined through stepwise logistic regression analysis. The results showed that LDL ≥ 100 mg/dl and CVD were strong predictors of statin use. Also, the odds of statin use were significantly higher in patients older than 60 years, male patients, patients with hypertension, HbA1c ≥ 8, and those with positive blood cultures (Table 4).

**Table 4:** Predictors of statin use among patients with COVID-19

Predictors	Statin group *n* = 200 *n* (%)	Non-statin group *n* = 347 *n* (%)	OR (95% CI)	*P* value
**Age > 60 years**	124 (72.0)	123 (35.4)	3.10 (2.10–4.30)	<0.001^**^
**Male sex**	151 (75.5)	189 (54.5)	2.70 (1.30–5.36)	<0.001^**^
**CVD**	97 (48.5)	51 (14.7)	3.80 (1.32–7.21)	<0.001^**^
**Hypertension**	128 (64.0)	149 (42.9)	2.20 (1.10–5.34)	0.02^**^
**HbA1c ≥ 8.0%**	78 (39.0)	79 (22.7)	2.40 (1.16–7.32)	0.01^**^
**LDL > 100 mg/dl**	152 (76.0)	95 (27.4)	11.1 (3.56–27.4)	0.001^**^
**Positive blood culture**	84 (42.0)	70 (20.2)	2.30 (1.12–6.89)	0.04^**^

Results of predictive stepwise logistic regression analysis **Significant

Table 5 indicates that statin use was associated with an 18% reduction in mortality risk among patients, although this finding was not statistically significant (adjusted OR = 0.82, 95% CI, 0.30–2.32). For other outcomes, a positive association was observed between mechanical ventilation and ICU admission (OR = 1.45), although not significant. Statin users had a slightly lower likelihood of a hospital stay exceeding 10 days, with an aOR of 0.93 (95% CI: 0.75–1.23); however, this finding was not statistically significant.

**Table 5:** The association between statin use and outcome-related factors among patients with COVID-19

Outcome factors	*n*	OR	(95% CI)	*P* value
**ICU admission** No statin use (*n* = 347) Statin use (*n* = 200)	63 (18.2) 57 (28.5)	1.00 1.45	Ref. 0.95-2.23	0.09
**Mechanical ventilation** No statin use (*n* = 347) Statin use (*n* = 200)	39 (11.2) 37 (18.5)	1.00 1.45	Ref. 0.89-2.41	0.13
**Mortality** No statin use (*n* = 347) Statin use (*n* = 200)	20 (5.8) 11 (5.5)	1.00 0.82	Ref. 0.30-2.21	0.15
**Days in hospital > 10 days** No statin use (*n* = 347) Statin use (*n* = 200)	107 (30.8) 59 (29.5)	1.00 0.93	Ref. 0.75-1.25	0.74^**^

*Adjusted by age category, sex, CVD, hypertension, LDL, HbA1c, and culture and sensitivity **Significant

## DISCUSSION

The findings revealed a gender disparity, with a higher proportion of men using statins. Previous studies also reported a gender gap in statin prescription rates despite similar cardiovascular risk profiles between men and women [21-23]. Factors such as differences in statin metabolism, side effects, and teratogenic effects on women of reproductive age may contribute to the lower prescription rates for women [23]. The study also found that statin therapy was not preferentially prescribed based on BMI, consistent with previous research emphasizing the importance of cardiovascular risk assessment beyond BMI alone [24].

Statin users had a significantly higher mean age, consistent with the understanding that age is a major determinant of CVD [25]. Furthermore, there was a significant association between statin use and cardiovascular risk factors such as established CVD, hypertension, and diabetes mellitus, supporting established guidelines. Statin therapy has been shown to reduce cardiovascular risk in patients with these risk factors and lower the risk of mortality [26].

The study findings revealed significant associations between the use of statins and LDL levels, HbA1c, and culture/sensitivity laboratory results. These findings are in line with previous studies that emphasized the effectiveness of statins in managing dyslipidemia and diabetes [27,28]. Numerous studies have demonstrated that statins are particularly effective in managing specific metabolic diseases [29]. As a result, statins have become the first-line therapy for reducing cholesterol levels [30]. In terms of blood culture, high-risk patients with underlying diabetes or atherosclerotic cardiovascular disease (ASCVD) who are on statins because of their risk category are more likely to develop secondary bacterial infections, resulting in positive culture results [31]. Thus, it is not surprising that participants with elevated levels of HbA1c, LDL, and positive culture results were more likely to be prescribed statins.

The predictive stepwise logistic regression analysis identified old age, gender, CVD, hypertension, and laboratory parameters (HbA1c, LDL, and positive culture) as independent predictor factors significantly associated with statin use, consistent with previous studies [27,28,32,33].

Our study found a negative association between statin use and mortality in this cohort, with a risk reduction of 18%. This aligns with the findings of Rossi *et al*. [34], who also reported a reduction in mortality with statin use, although the reduction was not statistically significant. Another study conducted by Peymani *et al*. [35] also reported a 24% decrease in COVID-19-related mortality among patients with COVID-19 using statins. In our study, statin use was associated with decreased COVID-19 disease severity, as evidenced by shorter hospital stays (less than 10 days). Findings from a retrospective study conducted in an elderly care facility indicated that the administration of statins to residents diagnosed with COVID-19 was linked to milder symptoms and improved clinical outcomes [17]. In contrast, a large retrospective study [36] conducted on 4,447 patients with COVID-19 hospitalized at the Johns Hopkins Hospital and affiliated hospitals reported a significant positive association between statin use and the risk of ICU admission, mechanical ventilation, and prolonged hospital stay (≥7 days).

### Limitations

The study presented comprehensive data on demographic, clinical, laboratory, and in-hospital outcomes, offering valuable insights into factors associated with statin use among hospitalized COVID-19 patients. However, several limitations should be considered when interpreting the findings. Firstly, the retrospective design utilized in this study limits its ability to establish causal relationships, although it identified associations between statin use and various clinical characteristics. Secondly, as with many retrospective studies, there is a potential risk of missing data and misclassification biases. Additionally, this study was conducted in only two centers, which limits its generalizability to the broader population. Therefore, more extensive longitudinal studies would be more appropriate to further explore the dynamic nature of these associations over time and assess causality.

## CONCLUSION

In summary, this study found that age, gender, CVD, hypertension, and laboratory parameters (HbA1c, LDL, and positive blood culture) influenced statin use in hospitalized COVID-19 patients. These findings contribute to our evolving understanding of statin use patterns and their associations with clinical characteristics in patients with COVID-19. The results are consistent with previous research and provide new insights into the association between statin therapy and hospitalization duration. It is crucial to address gender disparities, investigate the impact on various conditions, and understand the associations with laboratory parameters to optimize statin therapy and improve outcomes in COVID-19 management. This study could serve as a foundation for future research into the multifaceted effects of statin use and their implications in COVID-19 management. Finally, the findings of this study provide valuable insights into statin usage patterns and their associations with various clinical characteristics and in-hospital outcomes among hospitalized COVID-19 patients.

## Personal thanks

The author wishes to express gratitude to the IT staff at Ohud Hospital and King Salman Medical City (KSMC), Madinah, Saudi Arabia, and the directors of these institutions for providing the necessary support to conduct this study.
